# The role of both parents’ attachment pattern in understanding childhood obesity

**DOI:** 10.3389/fpsyg.2014.00791

**Published:** 2014-07-28

**Authors:** Claudia Mazzeschi, Chiara Pazzagli, Loredana Laghezza, Giulia Radi, Dalila Battistini, Pierpaolo De Feo

**Affiliations:** ^1^Department of Philosophy and Human Sciences, University of Perugia, PerugiaItaly; ^2^Department of Psychiatry, School of Clinical Psychology and Psychiatric Rehabilitation, PerugiaItaly; ^3^Health Lifestyle Institute, Centro Universitario Ricerca Interdipartimentale per l’Attività Motoria, University of Perugia, PerugiaItaly

**Keywords:** parents’ attachment, mother’s attachment, father’s attachment, childhood obesity, risk factor

## Abstract

Within the research area on the determinants of childhood obesity, a relatively new approach is the use of attachment theory to explore the mechanisms underlying children’s obesity risk, especially considered as emotion regulation strategies in parent–child relationship. Few are the empirical researches that have addressed this issue. The empirical investigations have used self-report measures to assess adult attachment. In attachment studies, the use of interview methods and/or performance-based instruments is advised to evaluate the entire range of possible adult attachment patterns and comprehensively explain the emotional strategies, correlates, and consequences of individual differences in attachment system functioning. The aim of this study was to explore the extent to which both parents’ attachment patterns serve as self-regulative mechanisms related to childhood overweight/obesity by the Adult Attachment Projective Picture System (AAP) in a sample of 44 mothers and fathers of children referred for obesity. Insecure attachment was found as a risk factor both for mothers and fathers. Also unresolved/disorganization was found to play a significant role in childhood obesity. The role of father’s attachment was explored and findings suggested considering it in etiology and treatment of childhood obesity.

## INTRODUCTION

The need to deal with obesity at an early stage has become a priority due to the increasing prevalence of childhood obesity (Hollinghurst et al., 2014). The multifactorial nature of pediatric obesity, which is based on both genetic and environmental factors, requires a broad framework (Silventoinen et al., 2010; Harrison et al., 2011). Observational and experimental evidence reveals persistent effects of the early environment on eating behavior and obesity risk, highlighting the central role parents play in the onset of childhood obesity (Anzman et al., 2010).

At different levels of analysis, the literature on childhood obesity has recently begun to investigate the contribution of family factors in terms of the “neglect construct” (Hughes et al., 2005). These include variables with respect to both parental and family domains in etiology and treatment (Berge, 2009; Guilfoyle et al., 2010; Monasta et al., 2010; Lehto et al., 2012; Mazzeschi et al., 2013; Bost et al., 2014). To date, most studies have primarily focused on the consumption behaviors and parental feeding styles that characterize “obesogenic families” (Davison and Birch, 2002), although a number of research gaps have been identified (Zeller et al., 2007; Faith et al., 2011). Initial studies have predominantly focused on maternal factors rather than adopting a perspective that includes both mothers and fathers, and studies on the relationship between determinants of pediatric obesity and paternal role are very scarce (Wake et al., 2007; Freeman et al., 2010). It is critical to identify the relevant psychosocial/parental characteristics of both parents to extend our understanding of how parenting behaviors and interpersonal environments affect children (Lindsay et al., 2006; Harrison et al., 2011) and to explore the mechanisms that relate parenting variables to childhood obesity (Frankel et al., 2012; Bost et al., 2014).

From a theoretical point of view, a number of studies place obesity within the framework of attachment theory (Golan and Crow, 2004; Frankel et al., 2012) due to its power to relate parents’ sensitivity in terms of the Internal Working Model (IWM) to the practice of feeding and regulating emotions in parents’ relationships with their children (Fiese et al., 2012). Bost et al. (2014) has noted that insecure attached parents may use negative regulations strategies in response to children’s distress, which has important consequences for interpersonal contexts involving food and the development of children’s eating behaviors. Dyadic exchanges between the parent and child might affect food consumption through their effect on emotion regulation and parental feeding practices (Fiese et al., 2012). The classification of attachment patterns has identified two dimensions: secure vs insecure and organized vs disorganized attachment (Liotti, 2000; Rutter et al., 2009). The secure dimension enables us to distinguish individual differences in attachment patterns, identifying secure attachment and two types of insecure attachment (dismissing and preoccupied). The second dimension, which refers to the organization of the attachment pattern, distinguishes organized attachment (secure, dismissing and preoccupied) from disorganized one. In disorganized attachment, there is a lack of coherence and integration in attachment strategies in the face of separation. In the secure attachment pattern, the attachment figure provides emotional feedback and the parent’s emotional availability enables the child to regulate his emotional experiences. Secure attached parents respond consistently and sensitively to the full range of infant emotions, thus allowing the infant to learn to self-regulate and develop strategies for managing increasing levels of arousal (Sloman et al., 2002). In contrast, insecure attached individuals tend to use regulatory strategies that are less flexible and adaptive. Insecure attached parents fail to regulate the children’s emotional experiences and fail to protect their children and make them feel safe (Bowlby, 1980; Main and Hesse, 1990). Specifically, insecure attached dismissing individuals rely on deactivating strategies to regulate affect (Cassidy and Kobak, 1988). A deactivating emotional strategy leads dismissing parents to restrict their range of emotional expression and to detach from interactions that might trigger negative feelings (Magai, 1999). In contrast, insecure attached preoccupied individuals employ hyper-activating processes that elicit children’s involvement (Mikulincer et al., 2003). In parenting, preoccupied parents inconsistently respond to their children’s needs and provide few emotional anchors to assist their children in regulating their emotional states (Sloman et al., 2002; DeOliveira et al., 2005). Along the organized/disorganized dimension, the disorganized attachment pattern is characterized by the absence of a unitary or coherent pattern or strategy (Hesse and Main, 2000). The disorganized attachment pattern is associated with attachment-related experiences of unresolved loss or trauma/abuse (Solomon and George, 1999). Disorganized individuals use affective strategies in which painful attachment-related memories are isolated in segregated systems and blocked from conscious thought (Bowlby, 1980). The segregated systems are defined as unresolved when they are not integrated or contained at the representational level (George and West, 2001, 2011, 2012). Hesse and Main (2000) has hypothesized that the unresolved parent behaves in a frightened or frightening way toward the child, causing the infant to experience dysregulated fear in relation to the caregiver, which produces disorganization in the child’s attachment strategy. Parental disorganized/unresolved attachment patterns are considered to be a strong risk factor for the child’s development of maladaptive self-regulation strategies (Solomon and George, 1999; DeOliveira et al., 2005). The dysregulated emotional response of insecure or disorganized parents puts them at risk for ineffective parenting behaviors, which are frequently observed in studies of parental and familial functioning of overweight/obese children (Zeller et al., 2007; Moens et al., 2009; Sleddens et al., 2011; Lehto et al., 2012).

The few empirical studies focusing on the relationship between attachment and obesity have provided interesting data. Two studies investigated the association between the quality of early mother–child attachment and the later risk of child obesity. In a cohort study, Anderson and Whitaker (2011) assessed attachment with the Toddler Attachment Sort and examined the association between the attachment style observed in the mother–child relationship at 24 months of age and obesity at 4½; years of age. Results comparing secure to insecure attachment styles suggested that the insecure mother–child attachment style in early childhood was a risk factor for obesity. Similar findings emerged when insecure and disorganized attachment styles were combined and compared with the secure attachment style. In a later study, the observed poor quality of the early mother–child attachment relationship was also associated with a higher prevalence of obesity in adolescence (Anderson et al., 2012).

Only two studies have focused on the quality of parental attachment and the risk of childhood obesity. Using the Attachment Style Questionnaire, a significant prevalence of the insecure attachment style was found in the mothers of 30 children with obesity (Trombini et al., 2003). Recently, Bost et al. (2014) applied a mediation model to the investigation of the role of caregivers’ attachment style on children’s food consumption. The caregivers’ attachment style was assessed with the Relationship Scales Questionnaire. The results indicated that insecure caregivers were more likely to report using negative emotion regulation strategies in response to their child’s distress, planned mealtimes less often, and allowed their children to watch more television. Attachment was also correlated with feeding style; insecure parents reported using emotion-feeding pressure practices more often than secure parents.

Although these research studies have begun to provide empirical support for the theoretical proposal that insecurity of attachment is a risk factor for child obesity, they present a number of limitations. The reported studies have exclusively focused on the maternal contribution to the risk of pediatric obesity. Moreover, the two studies that assessed mothers’ attachment patterns relied solely on self-report measures. In attachment studies, the use of interview methods and/or performance-based instruments is advised to evaluate the entire range of possible adult attachment patterns and comprehensively explain the emotional strategies, correlates, and consequences of individual differences in attachment system functioning. Jacobvitz et al. (2002) note that self-report measures of attachment elicit adults’ conscious appraisals of themselves in attachment relationships. Adults’ unconscious IWM and emotion-regulating processes, as well as the strategies developed by individuals to cope with the anxiety aroused by the narratives related to the attachment prompts, need to be explored to identify and overcome biases such as social desirability that can affect self-report results.

Based on the above considerations, the aim of this study was to explore the extent to which both parents’ attachment patterns serve as self-regulative mechanisms related to childhood overweight/obesity. Paternal and maternal attachment was assessed by the Adult Attachment Projective Picture System (AAP; George and West, 2001), with other parental measures for anxiety and depressive symptoms. This performance-based measure was chosen because it is the instrument of choice for assessing adults’ unconscious IWM and emotion-regulating processes. Maternal and paternal attachment patterns were analyzed separately to evaluate their different contributions to children’s overweight/obesity. To date, no study has investigated the role of each parents’ attachment pattern on child’s obesity using narrative instruments or considered the specific characteristics of different types of attachment insecurity patterns and disorganization. Consequently, the specific aims of the present study were to:

(1) investigate the role of both parents’ secure/insecure organized and of disorganized/unresolved attachment patterns on child’s overweight/obesity to extend the results of previous studies, which focused solely on the role of the mother’s insecure attachment style; confronting parents’ attachment pattern with data available from Italian normative sample
(2) analyze both parents’ different attachment patterns to identify particular emotional regulation strategies; and
(3) explore the unique contribution of each parents’ attachment pattern by employing both a two-way classification (secure vs insecure and organized/resolved vs disorganized/unresolved) and a four-way classification (secure, dismissing, preoccupied, and unresolved) to predict children’s weight.

According to previous studies, we expect that mothers of overweight/obese children exhibit a predominantly insecure attachment. Because of the lack of studies regarding fathers’ attachment and child obesity, no specific hypotheses are made. Considering attachment patterns as self-regulative mechanisms, we expect that mothers’ and fathers’ insecure attachment pattern predicts child BMI. Similarly, because of the fact that disorganized/unresolved attachment is a strong risk factor for dysregulation, we expect a prediction of the severity of child’s BMI, both for mothers’ and fathers’ disorganized/unresolved attachment pattern.

## MATERIALS AND METHODS

### SUBJECT RECRUITMENT AND THE RESEARCH CONTEXT

Data were collected at C.U.R.I.A.MO (Centro Universitario Ricerca Interdipartimentale per l’Attività Motoria), a center for childhood overweight–obesity treatment at the University of Perugia in Italy, approved by the local Ethics Committee (CEAS Umbria Region, HREC number 1/10/1633). The children, who ranged in age from 6 to 15 years, were referred to the center for overweight/obesity. Both caregivers provided written informed consent/assent after the families were briefed on the study; children provided verbal consent. The exclusion criteria were non-Italian-speaking caregivers, caregiver report of youth developmental delay or mental retardation or secondary overweight due to endocrinological diseases. Anthropometric measurement and psychological assessment were conducted by trained investigators, following standardized guidelines.

### SAMPLE

The sample consisted of the mothers and fathers of 44 children referred for obesity/overweight to the CURIAMO. All the subjects were Caucasian. Parent’s mean age was 42.2 years (SD = 4.7) for mothers and 45.6 (SD = 4.7) for fathers. The socio-economic status of the families was medium–low (mean = 36.39; SD = 10.72). The children mean age was11.35 years (SD = 2.9).

### MEASURES

#### Anthropometric measures

To compute BMI, participants were measured for height and weight at the first medical examination. Weight was measured using a digital scale (BC-420S MA; Tanita, Sindelfingen, Germany); height was measured three times using a stadiometer, and the average height based on these readings was used.

#### Parent measures

***State-Trait Anxiety Inventory Form Y.*** Anxiety was measured using the validated Italian version of the State-Trait Anxiety Inventory Form Y (STAI; Spielberger et al., 1970; Pedrabissi and Santinello, 1989). The STAI is a 40-item questionnaire measuring state (20 items) and trait (20 items) anxiety. Each scale consists of 20 items that are rated on a 4-point Likert scale ranging from 0 (none) to 4 (extremely high). The STAI assesses how respondents feel at the present moment (state) and how respondents generally feel (trait), indicating the extent to which the respondent experiences symptoms of anxiety. Total scores for trait and state anxiety range from 20 to 80. The STAI has demonstrated good internal consistency, test–retest reliability for the STAI Trait, sensitivity to the detection of stress for the STAI State, and convergent and discriminant validity (Spielberger, 1989; Barnes et al., 2002; Vautier, 2004). The STAI State (mothers: α = 0.90; fathers: α = 0.89) and the STAI Trait (mothers: α = 0.77; fathers: α = 0.81) demonstrated good internal consistency in the present study.

***Center for Epidemiologic Studies Depression Scale.*** This study used the Italian version of the Center for Epidemiologic Studies Depression Scale (CES-D; Fava, 1983), a 20-item, self-report measure originally designed for use in the general population that measures the frequency with which participants experienced specific symptoms of depression in the preceding week. The frequency of each symptom is rated on a scale ranging from 0 (rarely/none of the time, less than once a day) to 3 (most or all of the time, for 5–7 days). The CES-D test scores, which exhibit adequate internal consistency and test–retest reliability, correlate with clinical judgments and other self-report measures of depression, possess construct validity, and generate theoretically meaningful factors, such as depressed affect, somatic symptoms, and diminished positive affect (Radloff, 1977; Roberts, 1980; Fava, 1983; Iwata and Buka, 2002). The internal consistency of the scale in the present sample was good both for mothers (α = 0.88) and fathers (α = 0.86).

***The Adult Attachment Projective Picture System.*** The AAP (George and West, 2001, 2011, 2012) is an adult attachment classification system based on the analysis of a set of projective stimuli. The AAP consists of a semi-structured interview procedure in which the client is asked to narrate a story for each of seven attachment picture stimuli (George and West, 2001, 2012). The stimuli portray scenes associated with attachment distress, such as the threat of separation, loss, illness, death, and solitude. The scenes represent children or adults alone (alone pictures) or in potential attachment–caregiving relationships (dyadic pictures). Individuals are asked to narrate a story for each image that refers to “what is happening in the situation, what led up to the scene, what the characters are thinking or feeling, and what might happen next.” The AAP is audio-recorded and fully transcribed. Each AAP transcript is coded on seven scales grouped under the three major categories of discourse, content, and defensive processing. The AAP system identified also the individual’s distinctive patterns of attachment defensive processes (Bowlby, 1980; George and Solomon, 2008). Two forms of defensive processing are regarded as normative or organizing forms of defensive exclusion. Deactivation is defined as attempts to dismiss, cool off, or shift attention away from attachment events, individuals, or feelings in response to the picture stimuli. The second organizing form of defensive exclusion, cognitive disconnection, disconnects the elements of attachment from their source, thus undermining consistency and the ability to maintain a unitary view of events, emotions, and the individuals associated with them. A third form of defensive processing, segregated systems, describes a mental state in which painful attachment-related memories rooted in experiences of trauma or loss through death are isolated and blocked from conscious thought (Bowlby, 1980). Segregated systems are identified in the AAP by content connoting fear, odd/disturbing material and emptiness. Segregated systems represent the failure of normative defenses and develop when attachment figures do not protect and comfort children when they are frightened or threatened, particularly in the context of attachment figure loss. The scoring of these dimensions by the classification system produces four adult attachment patterns: secure (F), dismissing (Ds), preoccupied (E), and unresolved (U; George and West, 2011; Lis et al., 2011). This is generally termed a four-way attachment classification. These four attachment patterns can be grouped into two two-way attachment classifications: secure vs organized insecure (dismissing and preoccupied); and resolved (secure, dismissing, and preoccupied) vs unresolved (George and West, 2012).

The AAP provides researchers and clinicians with a construct-validated measure of attachment that preserves the emphasis on mental representation and defensive processes that is one of the primary features of attachment theory. For the purpose of establishing construct validity, the AAP has been validated using the Adult Attachment Interview (AAI; George et al., 1984/1985/1996), which exhibits a convergent agreement between the AAP and AAI for the four major attachment groups of 0.85 (kappa = 0.84, *p* < 0.001; George and West, 2001). The authors also found a strong inter-rater reliability of 0.86 (kappa = 0.79, *p* < 0.001). The AAP provides good test–retest reliability. In one study, George and West (2001) found that 84% of the 69 participants were classified in the same category after 3 months (kappa = 0.78, *p* < 0.001). The most recent review of AAP reliability and validity results is presented in George and West (2012). In the present study, two highly trained AAP raters performed an independent and blind coding of 20 randomly selected protocols. Inter-rater reliability was assessed using the Cohen’s (1990) kappa coefficient on AAP four-way attachment classification, and κ = 0.92. The remaining protocols were then scored independently.

### Data Analysis

Descriptive statistics for the samples were calculated as means and standard deviations or frequencies and percentages. Chi-square tests and Student’s *t*-tests for paired samples were used to compare the mothers and fathers on the psychological features of anxiety, depression, and the distribution of attachment patterns. To investigate the unique contribution of mothers’ and fathers’ attachment to child BMI, three ANOVAs were separately performed for mothers and fathers: one for secure vs insecure organized (F vs *D*s + *E*), one for each of the four attachment patterns (F vs Ds vs E vs U), and one for the resolved/organized vs disorganized/unresolved patterns (F + Ds + E vs U). A Bonferroni *post hoc* analysis was performed due to the multiple comparisons of parent attachment patterns in the four-way ANOVA. Effect size was measured using partial eta-squares, with small, medium, and large effects of 0.0099, 0.0588, and 0.1379, respectively (Cohen, 1988). Pearson correlation coefficients were used to assess the association between child BMI and maternal/paternal anxiety and depression. To investigate the extent to which the attachment patterns of mothers and fathers predicted child BMI, three linear regression analyses were separately performed for mothers and fathers to test the effect of security, each attachment pattern ,and disorganization on child BMI. The data were analyzed using SPSS version 21.0, and *p*-values of 0.05 or less were identified as statistically significant.

## RESULTS

The mean child BMI was 26.61 (SD = 4.6). Parent’s mean BMI was 24.96 (SD = 3.79) for mothers and 27.27 (SD = 3.45) for fathers. In terms of BMI classification (WHO), parents were overweight. With respect to anxiety and depressive mood, mothers’ and the fathers’ levels of state anxiety (mothers: *M* = 38.97, SD = 8.49; fathers: *M* = 38.31, SD = 8.65), trait anxiety (mothers: *M* = 40.88, SD = 6.20; fathers: *M* = 40.38, SD = 6.69) and depressive mood (mothers: *M* = 14.35, SD = 10.03; fathers: *M* = 12.30, SD = 9.16) did not reach clinical level (cut-off ≥ 16). Student’s *t*-tests for paired samples revealed no significant differences between mothers and fathers for state anxiety (*t*_(44)_ = 0.183, *p*= 0.856), trait anxiety (*t*_(44)_ = 0.415, *p*= 0.680) or depressive mood (*t*_(44)_ = 0.234, *p* = 0.324).

Frequencies and percentages were calculated for secure and insecure attachment, for the four attachment patterns, and for the resolved/organized vs unresolved/disorganized patterns between mothers and fathers (**Table 1**). Chi-square analyses revealed significant different distributions between parents for the secure and insecure attachment patterns (χ^2^ = 5.236, *df* = 1, *p*= 0.022) and for the four attachment patterns (χ^2^ = 18.17, *df* = 9, *p*= 0.032). Qualitative analyses revealed that mothers exhibited significantly higher frequencies of the preoccupied attachment pattern compared to fathers, who exhibited higher frequencies of the dismissing pattern. There were no significant differences in the distribution of the resolved/organized and unresolved/disorganized patterns (χ^2^ = 2.078, *df* = 1, *p*= 0.14).

**Table 1:** Frequencies and percentage for mothers and fathers on AAP attachment patterns.

		Secure	Insecure	*F*	*D*s	*E*	*U*	Resolved	Unresolved
Mothers	*N*	10	34	10	8	15	11	33	11
	%	*30.3%*	*69.7%*	*22.8%*	*18.2%*	*34%*	*25%*	*75%*	*25%*
Fathers	*N*	7	36	7	19	5	12	32	12
	%	*24.3%*	*75.7%*	*16%*	*42%*	*13%*	*29%*	*72.7%*	*27.3%*

To compare mothers’ and fathers’ attachment patterns with a normative Italian sample, a Chi-square analysis was conducted using data from a meta-analysis (Cassibba et al., 2013). Findings revealed a significant different distribution for mothers (χ^2^ = 24.49, *df* = 9, *p*= 0.004) for the four-way pattern classification. Only the three-way distribution was available for fathers, and no significant association was found (χ^2^ = 3.89, *df* = 4, *p*= 0.421). The analyses of variance (ANOVAs) revealed that both mothers’ and fathers’ attachment patterns had a significant effect on child BMI that was different for the two AAP patterns (secure vs insecure: *F* = 36.806, *p*= 0.001, η^2^= 0.429) and the four AAP patterns (*F* = 23.139, *p*= 0.001, η^2^= 0.547). The resolved/organized vs unresolved/disorganized pattern (*F* = 43.353, *p*= 0.001, η^2^= 0.386) also differentiated child BMI (**Table 2**).

**Table 2:** Analysis of variance (ANOVA) for mothers’ and fathers’ attachment on child’s BMI.

	Mothers											
	*S* vs *I*				Four-way				*R* vs *U*			
	*F*	*df*	*p*	η^2^	*F*	*df*	*p*	η^2^	*F*	*df*	*p*	η^2^

BMI	36.18	1	0.000	0.415	13.88	1	0.000	0.523	25.01	1	0.000	0.385

	Fathers											
	***S*** **vs** ***I***				**Four-way**				***R*** **vs** ***U***			
	***F***	***df***	***p***	η^**2**^	***F***	***df***	***p***	η^**2**^	***F***	***df***	***p***	η^**2**^

BMI	38.88	1	0.000	0.481	12.95	1	0.000	0.590	18.92	1	0.000	0.396

*Effect size measured using partial η^2^= 0.001 constitutes a small effect, 0.06 a medium effect, and 0.14 a large effect.*

Children whose mothers exhibited a secure attachment pattern had lower BMIs than children whose mothers exhibited dismissing, preoccupied, or unresolved attachment patterns (**Table 3**). Based on the AAP classification, children whose mothers exhibited dismissing and preoccupied patterns had higher BMIs than children with secure attached mothers. The BMI of children with mothers who exhibited unresolved attachment was significantly higher than the BMI of children with secure attached mothers. The same trend was also found for fathers (**Table 3**).

**Table 3:** Means and standard deviations of child’s BMI for mothers’ and fathers’ attachment.

	Mothers			Fathers		
	*M*	SD	*Post hoc*^1^	*M*	SD	*Post hoc*^1^
Secure	23.47	2.35		22.40	0.52	
Insecure	28.10	2.55		27.94	2.77	

Secure	24.06	2.77	F < Ds = E < U	22.40	0.54	F < Ds = E < U
Dismissing	27.57	3.04		27.69	3.01	
Preoccupied	28.53	1.92		28.75	2.22	
Unresolved	33.30	4.76		33.30	4.76	

Resolved	27.01	3.07		26.68	3.42	
Unresolved	33.30	4.77		33.92	4.07	

^1^F, secure; Ds, dismissing; E, preoccupied; U, unresolved.

The correlation analysis did not identify significant relationships between child BMI and parental state anxiety (mothers: *r*= -0.041, *p* = 0.718; fathers: *r*= -0.102, *p* = 0.540), trait anxiety (mothers: *r*= 0.085, *p* = 0.455; fathers: *r*= 0.018, *p* = 0.915) or depressive mood (mothers: *r*= -0.024, *p* = 0.834; fathers: *r* = 0.116, *p* = 0.487). Study data were consistent with a recent systematic review and meta-analyses (Weng et al., 2012).

Three multivariate linear regressions were performed to measure the unique contribution of mothers’ and fathers’ attachment to child BMI (**Table 4**). In the first model, mothers (*F* = 16.41, *p* = 0.000) and fathers (*F* = 14.19, *p* = 0.000) attachment security both negatively predicted child BMI. In the second model, which included the four patterns, only the secure and the unresolved patterns made a significant contribution (mothers: *F* = 10.19, *p* = 0.000; fathers: *F* = 9.96, *p* = 0.000), while the dismissing and preoccupied patterns were excluded. In the third model, disorganized/unresolved attachment predicted child BMI for both mothers (*F* = 25.01, *p* = 0.000) and fathers (*F* = 18.92, *p* = 0.000).

**Table 4:** Linear multivariate regression for mothers and fathers attachment patterns on child’s BMI.

	*R*	*R*^2^	β	*t*	*p*
**Mothers**					
*S* vs *I*	0.624	0.389	-0.624	-4.29	0.000
Four-way	0.671	0.450			
Secure (F)			-0.578	-4.84	0.000
Unresolved (U)			0.401	3.36	0.002
*R* vs *U*	0.620	0.369	0.620	5.00	0.000

**Fathers**					
*S* vs *I*	0.593	0.352	-0.593	-3.97	0.000
Four-way	0.770	0.593			
Unresolved (U)			0.505	5.82	0.000
Secure (F)			-0.457	-5.27	0.000
*R* vs *U*	0.629	0.375	0.629	4.35	0.000

## DISCUSSION

This study contributes to the broader debate on the role of family factors in childhood obesity, specifically the role of parent attachment, by empirically investigating the characteristics and relationship of parent’s attachment to child BMI in a sample of parents of children with overweight/obesity. As noted previously, few empirical papers have addressed this issue, and earlier findings, while promising, require further investigation. In this paper, the AAP was chosen for its power to capture attachment strategies and in order to overcome the limitations of the self-report measures (Jacobvitz et al., 2002) used in previous empirical research (Trombini et al., 2003; Bost et al., 2014). AAP produces four patterns (a four-way classification) near to secure vs insecure and organized/resolved vs disorganize/unresolved (George and West, 2012).

According to our hypothesis, mothers of overweight/obese children exhibit a predominantly insecure attachment as earlier literature showed (Trombini et al., 2003; Bost et al., 2014). This study also investigated fathers and found that they also exhibited insecure attachment patterns. To our knowledge, no previous studies had investigated the relationship between fathers’ attachment and childhood obesity because the mother generally served as informant (Bost et al., 2014). In this sample of parents of overweight/obese children, attachment security was not the main feature neither for mothers nor for fathers. In the distribution of attachment of the Italian non-clinical mothers, the security emerges as the main feature (Cassibba et al., 2013).

With regard to the first aim, in the four specific patterns of attachment identified by the AAP, the mothers of the study were categorized as prevalently preoccupied, unresolved and secure, and fathers were categorized as dismissing, unresolved, and secure. The comparison of these patterns of parental attachment with the data available for both Italian normative samples and clinical/at risk samples (Cassibba et al., 2013) indicated that for the four-way classification, the mothers in the sample were more likely to exhibit the preoccupied and unresolved patterns compared to the non-clinical mothers. Moreover, this distribution was similar to the distribution of the clinical/at risk Italian normative sample, suggesting that mothers of overweight/obese children were more similar to mothers in the normative risk group than to mothers in the non-clinical normative sample. For the four-way classification, fathers were more likely to be classified as dismissing. The comparison of these findings with normative data indicates the need to also examine the role of the father and paternal attachment in childhood obesity.

With respect to the second aim, on the distinction between organized/resolved and disorganized/unresolved patterns of attachment, mothers and fathers in the study sample did not differ significantly. Mothers and fathers in the study were primarily classified as organized, although there was a higher prevalence of disorganized/unresolved attachment in the study sample compared to the normative sample (Cassibba et al., 2013). Findings indicated that parents of overweight/obese children differed from the non-clinical normative sample for the prevalence of disorganized/unresolved attachment and were more similar to the clinical/at risk normative sample. Moreover, the distribution of parental attachment categories was different for mothers and fathers.

According to George and West (2012), the preoccupied, dismissing, and unresolved patterns of attachment that were seen in the present study for mothers and fathers represent different ways of coping with attachment needs and intimacy. Shaver and Mikulincer’s (2002) model claims that these three patterns represent different types of affect regulation in parenting. The preoccupied pattern of attachment relies on hyper-activating strategies (Cassidy and Kobak, 1988) at the cost of keeping the attachment system constantly activated and alert to the possibility of separation. Parents with this type of attachment pattern tend to respond inconsistently, be overprotective and interfere with their children’s needs (Cassidy and Kobak, 1988). The dismissing strategy relies on deactivating processes that produce an emotional “distance” from attachment-activating experiences and emphasize self-reliance. Parents with this type of strategy keep the attachment system in check at the cost of emotional openness; they have low levels of emotional involvement in close relationships and deny basic needs. The disorganized/unresolved pattern lacks a coherent and unique strategy; parents with this type of pattern risk becoming deeply disturbed by their unresolved experiences and unable to alleviate their child’s distress or develop their child’s resources. Mothers and fathers of overweight/obese children exhibit affect regulation strategies that conflict with secure attachment patterns, which might be related to the child’s level of overweight/obesity.

The present study was able to identify some associations between parental attachment and childhood overweight/obesity. Mothers’ and fathers’ insecure attachment predicted child BMI. Children with insecure attached mothers exhibited higher BMIs. Similarly, fathers’ insecure attachment was related to the severity of the problem. Consistent with the findings of Bost et al. (2014), insecurely attached parents may use negative regulation strategies in response to child distress, with important consequences for interpersonal contexts involving food and the development of children’s eating behaviors. Findings for our sample indicated that secure attachment appeared to be a protective factor because children of secure mothers and fathers exhibited the lowest BMIs.

Regarding the role of the specific patterns of attachment to child BMI, study findings indicated that the dismissing and preoccupied patterns of attachment did not contribute differently to child BMI for either mothers or fathers. It was the overall aspect of insecure attachment that differentiated between mothers and fathers contribution to child BMI. The two insecure patterns (Ds and E) did not differentially contribute to the severity of the child’s obesity. Moreover, according to our hypothesis, the unresolved/disorganized pattern contributed significantly to child obesity for both mothers and fathers and was related to the severity of the problem. Parents’ unresolved attachment is held to be a strong risk factor for the child’s development of maladaptive self-regulation strategies (Solomon and George, 1999; DeOliveira et al., 2005). This finding was confirmed by the comparison between child BMI and resolved/organized attachment, which found a significant difference in the severity of the child’s overweight/obesity for both mothers and fathers. Moreover, both maternal and paternal disorganized attachment positively predicted the severity of the child’s obesity. Study findings suggest that parental attachment potentially contributes to pediatric obesity, identifying an additional family component to the multiple sources of childhood obesity (Monasta et al., 2010). Specifically, study findings confirm evidence from earlier empirical research, which found that insecure and unresolved/disorganized parental attachment were risk factors for higher levels of childhood food intake. The present study provides the additional information that this relationship holds for both mothers and for fathers, although neither the dismissing nor preoccupied attachment patterns exerted a special or unique contribution to the severity of the child’s weight problem. Another contribution was the finding that unresolved/disorganized attachment was a powerful risk factor for both mothers and fathers, confirming that the dysregulation of affect that characterizes this pattern of attachment might interfere with the parents’ understanding of the child’s needs and affect food intake.

Some limitations of the study should be noted. First, the sample size was not large and belonged to a medium–low socioeconomic status. Findings need to be used considering the small size of the sample and its socio-economical features. Because the specific focus of this paper was the investigation of the separate contribution of mothers’ and fathers’ attachment, parents’ BMI was not used in the analysis. A previous work (Mazzeschi et al., 2013) showed the existence of a relationship between child and parent BMI. Further investigation are needed in order to put together, trough more complex model, the joint contribution of these variables in order to consider a broader range of possible factors influencing child BMI. A third limitation regards the fact that the study did not employ a normal weight control group, and comparisons were based on data from an Italian meta-analysis (Cassibba et al., 2013) that was primarily conducted using the distribution of categories derived from the use of AAI. If a high convergent agreement between AAP and AAI has been devised (George and West, 2012), some differences could be advocated with specific regard to the unresolved pattern of attachment and the possibility for AAP to assess different kinds of trauma or is severity (Delvecchio et al., 2013). More studies in this direction are needed, to better understand the separate and joint contributions of parents’ attachment to childhood obesity. In spite of these limitations, findings from this study suggest how important is the role of parental attachment in child obesity and underline the need to be considered in order to develop effective treatment plans.

## AUTHOR CONTRIBUTIONS

Claudia Mazzeschi: substantial contribution to the conception of the work, to the research design, to data collection and interpretation; drafting the work; final approval of the version. Agreement to be accountable for all aspects of the paper. Chiara Pazzagli: substantial contribution to the research design, to data interpretation; drafting the work; final approval of the version; agreement to be accountable for all aspects of the work. Agreement to be accountable for all aspects of the paper. Loredana Laghezza: contribution to data collection; substantial contribution to data analysis; drafting the paper; approval of the version to be published. Agreement to be accountable for all aspects of the paper. Giulia Radi: contribution to data collection. Agreement to be accountable for all aspects of the paper. Dalila Battistini: contribution to data collection. Agreement to be accountable for all aspects of the paper. Pierpaolo De Feo: contribution to data interpretation; revising the work; finally approval of the version. Agreement to be accountable for all aspects of the paper.

## Conflict of Interest Statement

The authors declare that the research was conducted in the absence of any commercial or financial relationships that could be construed as a potential conflict of interest.
